# The complete chloroplast genome of *Glehnia littoralis*, an Endangered medicinal herb of Apiaceae family

**DOI:** 10.1080/23802359.2018.1507638

**Published:** 2018-10-30

**Authors:** Yifeng Zhou, Maolin Geng, Mimi Li

**Affiliations:** a Institute of Botany, Jiangsu Province and Chinese Academy of Sciences, Nanjing, China;; b The Jiangsu Provincial Platform for Conservation and Utilization of Agricultural Germplasm, Nanjing, China

**Keywords:** Chloroplast genome, cp, *Glehnia littoralis*, conservation genetics, Apiaceae

## Abstract

*Glehnia littoralis* is an important medicinal herb disjunctly distributed at sandy beaches in eastern Asia and western North America. The complete chloroplast genome sequence of *G. littoralis* is 147,552 bp in length and structurally divided into four distinct regions: two copies of inverted repeat of 18,365 bp separated by a large single copy (LSC) of 93,277 bp and a small single copy (SSC) of 17,545 bp. A total of 129 genes are annotated including 85 protein-coding genes, 36 tRNA gene, and eight rRNA genes. Phylogenetic relationship revealed that *G. littoralis* is closely related to *Angelica dahurica* in Apiaceae.

*Glehnia littoralis* Fr. Schmidt ex Miq., a member of carrot family (Apiaceae), is a typical coastal species distinctive distributed in eastern Asia and western North America disjunctly. Its dried roots and rhizomes, generally called Bei-Sha-Shen, are important traditional Chinese medicine for treating lung diseases. Wild *G. littoralis* is rare and Endangered at present because of habitat loss and over-exploitation. The population in Jiangsu province was even local extinction (Song et al. 2013). It has been listed as a second-level protected species by Chinese government and a Critically Endangered species (CR) in China Biodiversity Red List—Higher Plants. However, there is no comprehensive genetic researches were conducted for *G. littoralis* due to lack of genomic resources until now. Therefore, we assembled the complete chloroplast (cp) genome of *G. littoralis* based on Illumina paired-end sequencing. Our study will provide a valuable plastid genomic resource for population genetics studies.

The individuals of *G. littoralis* were collected from Xichong Beach, Shenzhen (22°28′39.11″ N, 114°31′54.72″ E). The voucher specimen has been deposited in the Herbarium of Institute of Botany, Jiangsu Province and Chinese Academy of Sciences (NAS). The total genomic DNA was extracted from fresh leaves using Plant Genomic DNA Kit DP305 (Tiangen, Beijing) and sequenced on Illumina Hiseq TM 4000 platform (San Diego, CA). The raw data were assembled after filtering out low-quality sequences according to the protocol of Hahn et al. (2013). The complete cp genome was annotated with DOGMA (Wyman et al. 2004) and adjusted start and stop codons manually. The circular cp genome map was drawn using OGDRAW (Lohse et al. 2013).

The complete cp genome sequence of *G. littoralis* (MH142518) was 147,552 bp in length. It harbored a typical quadripartite and circular structure including a large single copy (LSC) of 93,277 bp and a small single copy (SSC) of 17,545 bp separated by a pair of inverted repeat (IRa and IRb) of 18,365 bp. In the chloroplast genome, 114 unique genes were identified, containing 80 protein-coding genes, 30 transfer RNA (tRNA), and four ribosomal RNA (rRNA). Sixteen genes contained one intron, including 10 protein-coding genes, and six tRNA. *Ycf3* and *clpP* had two introns. The GC content of whole cp genome was 37.5%.

To explore the divergence hotspot regions within *G. littoralis*, the cp genome sequence was compared to another available *G. littoralis* (KT153022) (Lee et al. 2015) comprehensively at the genome scale. We detected 47 mutations (26 SNPs and 21 indels), 38 in the LSC region, five in the SSC region, and four in the IR regions. All these sites would provide potential molecular markers for further conservation genetics and phylogeographic studies of *G. littoralis*.

An alignment was generated in Geneious 10 (Kearse et al. 2012) using the MAFFT plugin (Katoh and Standley 2013) to reconstruct molecular phylogenetic tree within Apiaceae with the Neighbor-Joining (NJ) method that employed in MEGA 6 (Tamura et al. 2013). Two species in Araliaceae were chosen as outgroups. As a result, individuals of *G. littoralis* formed a well-supported monophyletic clade closely related to *Angelica dahurica* (Figure 1).

**Figure 1.**
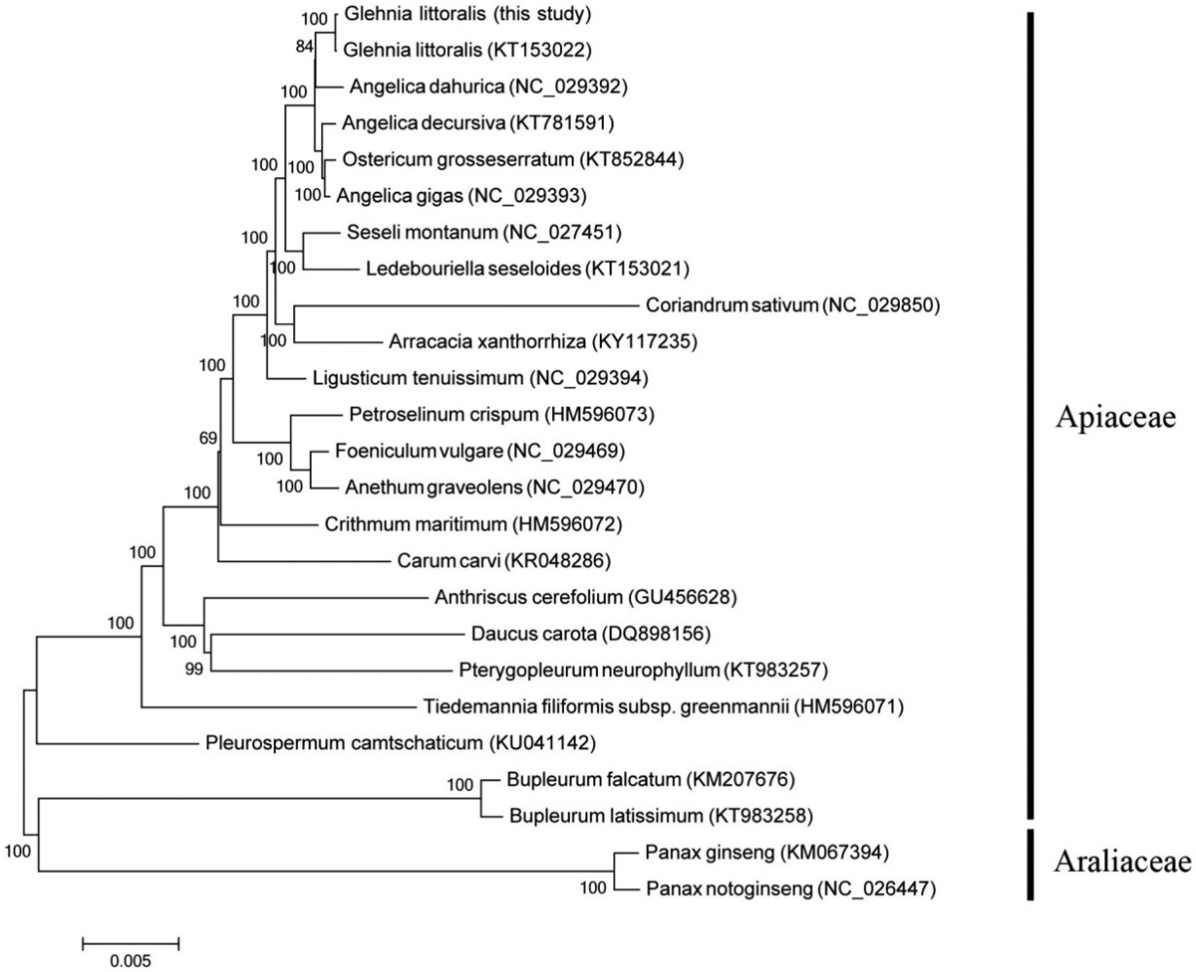
Phylogenetic relationships of 23 species within Apiaceae based on the Neighbor-Joining (NJ) method with *Panax notoginseng* (NC_026447) and *Panax ginseng* (KM067394) as outgroups.
